# Comparative Transcriptome Analysis Shows Conserved Metabolic Regulation during Production of Secondary Metabolites in Filamentous Fungi

**DOI:** 10.1128/mSystems.00012-19

**Published:** 2019-04-16

**Authors:** Jens Christian Nielsen, Sylvain Prigent, Sietske Grijseels, Mhairi Workman, Boyang Ji, Jens Nielsen

**Affiliations:** a Department of Biology and Biological Engineering, Chalmers University of Technology, Gothenburg, Sweden; b Department of Biotechnology and Biomedicine, Technical University of Denmark, Lyngby, Denmark; c Novo Nordisk Foundation Center for Biosustainability, Technical University of Denmark, Lyngby, Denmark; University of California, Berkeley

**Keywords:** comparative transcriptomics, cell factories, filamentous fungi, secondary metabolism

## Abstract

Filamentous fungi possess great potential as sources of medicinal bioactive compounds, such as antibiotics, but efficient production is hampered by a limited understanding of how their metabolism is regulated. We investigated the metabolism of six secondary metabolite-producing fungi of the *Penicillium* genus during nutrient depletion in the stationary phase of batch fermentations and assessed conserved metabolic responses across species using genome-wide transcriptional profiling. A coexpression analysis revealed that expression of biosynthetic genes correlates with expression of genes associated with pathways responsible for the generation of precursor metabolites for secondary metabolism. Our results highlight the main metabolic routes for the supply of precursors for secondary metabolism and suggest that the regulation of fungal metabolism is tailored to meet the demands for secondary metabolite production. These findings can aid in identifying fungal species that are optimized for the production of specific secondary metabolites and in designing metabolic engineering strategies to develop high-yielding fungal cell factories for production of secondary metabolites.

**IMPORTANCE** Secondary metabolites are a major source of pharmaceuticals, especially antibiotics. However, the development of efficient processes of production of secondary metabolites has proved troublesome due to a limited understanding of the metabolic regulations governing secondary metabolism. By analyzing the conservation in gene expression across secondary metabolite-producing fungal species, we identified a metabolic signature that links primary and secondary metabolism and that demonstrates that fungal metabolism is tailored for the efficient production of secondary metabolites. The insight that we provide can be used to develop high-yielding fungal cell factories that are optimized for the production of specific secondary metabolites of pharmaceutical interest.

## Abstract (precis)

Secondary metabolites are a major source of pharmaceuticals, especially antibiotics. However, the development of efficient processes of production of secondary metabolites has proved troublesome due to a limited understanding of the metabolic regulations governing secondary metabolism. By analyzing the conservation in gene expression across secondary metabolite-producing fungal species, we identified a metabolic signature that links primary and secondary metabolism and that demonstrates that fungal metabolism is tailored for the efficient production of secondary metabolites. The insight that we provide can be used to develop high-yielding fungal cell factories that are optimized for the production of specific secondary metabolites of pharmaceutical interest.

## INTRODUCTION

Filamentous fungi are economically and ecologically important microorganisms and serve diverse applications in industrial biotechnology. Some of the key industrial processes utilizing these organisms include the production of pharmaceuticals (1), bulk chemicals (2), and enzymes (3) and the manufacture of fermented food products (4). One important characteristic of many filamentous fungi is their ability to secrete bioactive compounds, called secondary metabolites, which are renowned for their pharmaceutical properties, e.g., as antibiotics, but also for their toxic effects, which are major health concerns when they contaminate food and feed (5). The fungal genus *Penicillium* is especially well-known for the production of many secondary metabolites (6, 7).

The large-scale industrial production of secondary metabolites is one of the greatest success stories of industrial biotechnology. The yield and titers of penicillin have been improved by orders of magnitude through decades of random mutagenesis and selection of Penicillium chrysogenum mutants (8, 9). Conversely, only limited success has been achieved through the use of rational metabolic engineering strategies to improve secondary metabolite production in fungi (10, 11). A major explanation for this is the limited understanding of the metabolic processes that govern the production of secondary metabolites in filamentous fungi.

Secondary metabolism is strongly connected to primary metabolism, in the sense that precursors and cofactors for secondary metabolites are derived from processes in the central carbon metabolism. The two main classes of secondary metabolites in fungi are polyketides (PKs), which are derived from short-chain acyl coenzyme A (acyl-CoA) units and which are synthesized by polyketide synthases (PKS), and nonribosomal peptides (NRPs), which are derived from amino acids and which are synthesized by nonribosomal peptide synthetases (NRPSs). Additionally, secondary metabolite pathways use cofactors, such as ATP and reducing power in the form of NADPH derived from energy metabolism. The link between the generation of secondary metabolite precursors and secondary metabolites is, however, poorly understood. Since secondary metabolites often are induced under suboptimal growth conditions, e.g., after depletion of a nutrient source (12), precursor units might not be synthesized *de novo* through glycolytic catabolism of carbon sources but, rather, might be synthesized through the degradation of stored macromolecules. The degradation of fatty acids and branched-chain amino acids (BCAAs) has been suggested to contribute to the acetyl-CoA supply for certain PKs in *Aspergillus* species (13), but a more comprehensive overview is needed.

Here, we analyzed secondary metabolism at the transcriptional level during stationary phase of batch fermentation experiments of secondary metabolite-producing *Penicillium* species (7, 14). The aim was to elucidate the link between primary and secondary metabolism and to improve the understanding of how the metabolism of native secondary metabolite producers is wired for the efficient production of secondary metabolites. We conducted a comparative transcriptome analysis of six *Penicillium* species cultivated in a defined medium (DM) and a complex medium (CM) with the objective to identify orthologous protein groups representing conserved metabolic features in these species.

## RESULTS

### Experimental setup for comparative transcriptomics of *Penicillium*.

**FIG 1:**
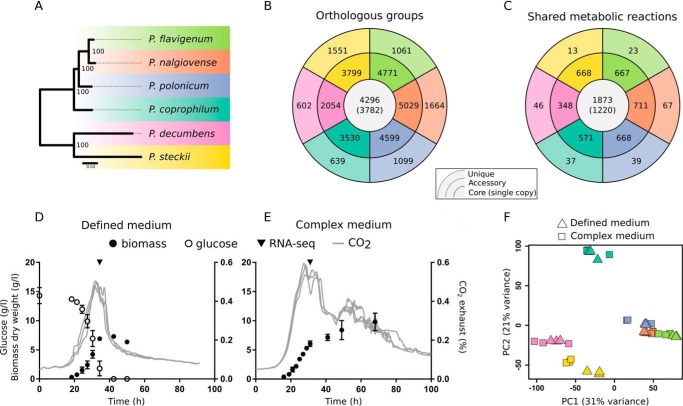
Overview of study and comparative analysis of *Penicillium* species. (A) Phylogenetic tree of the species investigated in this study. The colors in the tree serve as a legend for the entire figure. (B and C) Numbers of genes (B) and reactions (C) within each of the species that are shared by all species (core), that are shared by a subset of species (accessory), or that are specific to one species (unique). (D and E) Example growth profile of Penicillium flavigenum in defined medium (D) and complex medium (E), indicating the time point of the RNA-seq sample (generated based on previously published data [14]). (F) Principal-component analysis of the six *Penicillium* species based on the normalized expression of single-copy core genes.

Six *Penicillium* species (P. flavigenum, P. nalgiovense, P. polonicum, P. coprophilum, P. decumbens, and P. steckii) whose genomes were recently sequenced were subject to a comparative transcriptome analysis in order to assess evolutionarily conserved patterns of expression, with a specific emphasis on secondary metabolite biosynthesis. The species were selected because they represent the phylogenetic diversity within the *Penicillium* genus (15) (Fig. 1A) and because studies have highlighted their prolific capabilities for the biosynthesis of secondary metabolites (6, 14), as well as a genomic potential for additional secondary metabolite production (7).

For all species, protein-coding genes were clustered into orthologous groups (OGs). Among the 4,296 genes representing the core genome, 3,782 genes were present only in a single copy in the genomes (Fig. 1B), and these formed the basis for a phylogenetic assessment (Fig. 1A). Our previously published genome-scale metabolic models of the *Penicillium* species (16) served as an annotation of metabolic genes as defined in the MetaCyc database (17). We found that 582 single-copy OGs were part of the core metabolism and that 1,220 metabolic reactions were present in all metabolic networks (Fig. 1C). These core reactions were significantly depleted for reactions involved in secondary metabolism (adjusted *P = *5e−9, hypergeometric test), in particular, alkaloid and terpenoid biosynthesis. Conversely, all other parts of metabolism (as defined in the MetaCyc database), except for inorganic nutrient metabolism, were significantly enriched in the core genome fraction (adjusted *P < *0.05, hypergeometric test).

All species were cultivated in batch cultures in two different media: one defined medium (DM) for *Penicillium* containing glucose and ammonium and one industrially relevant complex medium (CM) based on yeast extract, sucrose, and nitrate. Since the aim was to investigate secondary metabolism, which is often induced under suboptimal growth conditions (12, 18), samples for transcriptome analysis were collected in the stationary phase several hours after CO_2_ production had peaked, indicating that the preferred carbon source had been depleted from the two carbon-limited media (Fig. 1D and E; see also Fig. S1 in the supplemental material).

10.1128/mSystems.00012-19.1 FIG S1 Growth profiles of *Penicillium* species in bioreactor fermentations. Samples for RNA-seq analysis were collected from previously published bioreactor cultivations (14). Triangles indicate the time point where biomass for transcriptome analysis was collected. The figure was generated based on previously published data (14). Download FIG S1, PDF file, 0.2 MB. Copyright © 2019 Nielsen et al. 2019 Nielsen et al. This content is distributed under the terms of the Creative Commons Attribution 4.0 International license.

To assess the global differences in gene expression between the species, we performed a principal-component analysis (PCA) based on single-copy core genes (Fig. 1F). The clustering of the samples in the PCA largely reflected the phylogeny of the species (Fig. 1A), indicating that the regulation of the core genes is evolutionarily related across species, in agreement with previous observations in different yeast species (19). In contrast, the medium composition had a minor impact on the clustering of the samples. Since batch effects could influence the comparison of expression levels across species, we tested the robustness of the PCA by evaluating multiple different strategies for normalization of the data, but in all cases, similar clustering patterns were observed (Fig. S2).

10.1128/mSystems.00012-19.2 FIG S2 Principal-component analysis (PCA) based on expression levels of orthologous genes in six *Penicillium* species using different strategies for normalization of the data. All normalization strategies resulted in a similar grouping of the samples in the PCA. (A) Log-transformed expression. (B) Z-score-transformed expression. (C) Rank-transformed expression. (D) Minimum/maximum normalization of expression, grouping it between 0 and 1. Squares indicate samples from the defined medium, and triangles indicate samples from the complex medium. Download FIG S2, PDF file, 0.03 MB. Copyright © 2019 Nielsen et al. 2019 Nielsen et al. This content is distributed under the terms of the Creative Commons Attribution 4.0 International license.

### Transcriptional landscape of *Penicillium*.

**FIG 2:**
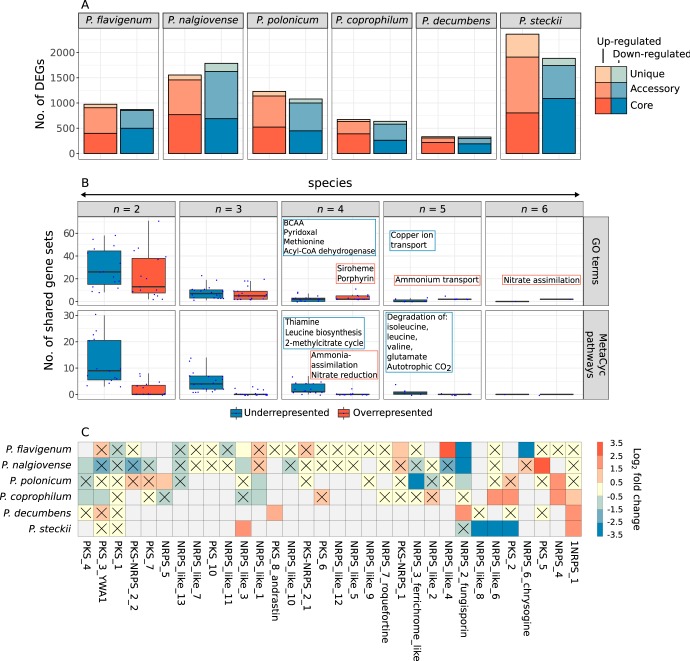
Main transcriptional differences between the media. (A) Number of genes differentially expressed in complex medium versus defined medium. (B) Gene set enrichment analysis identifying cellular processes shared by at least two species. Each data point in the box plots represents a gene set enriched by a combination of *n* species and grouped between up- and downregulated genes. The main shared processes are written in the frames. The boxes in each box plot represent the first, second, and third quartiles, and the whiskers extend to the lowest/highest values that are within 1.5 the interquartile range (IQR). (C) Effect of the media on the expression of secondary metabolite backbone genes that were present in at least two species. Cells in the heat map marked with a cross represent backbone genes that were not significantly differentially expressed. Upregulation and downregulation refer to expression levels in complex medium compared to that in defined medium. Gray cells indicate backbone genes not detected in the species.

Differentially expressed genes (DEGs) in CM relative to DM were identified in each species (adjusted *P < *0.05). A great variation in the number of DEGs per species was observed, ranging from 327 upregulated genes and 331 downregulated genes in P. decumbens to 2,363 upregulated genes and 1,885 downregulated genes in P. steckii (Fig. 2A). Interestingly, among the metabolic DEGs, secondary metabolism was the most affected part of metabolism among both up- and downregulated genes, thus emphasizing the differential effects of the media (Fig. S3). Among the core DEGs, we found only two genes that were upregulated in CM in all six species, and these were a nitrate reductase (ortholog, NIAD in Aspergillus nidulans) and an ammonium uptake transporter (ortholog, MEAA in A. nidulans). Both genes have been shown to respond to nitrate availability: NIAD reduces nitrate to nitrite intracellularly and is known to be upregulated in response to hypoxia (20), while MEAA is a low-affinity ammonium transporter which has proven to be upregulated under nitrogen starvation and induced by nitrate (21). No shared DEGs were downregulated in all species.

10.1128/mSystems.00012-19.3 FIG S3 Functional grouping of DEGs related to metabolism. Up- and downregulated DEGs from each species are annotated based on the MetaCyc annotation of the genome-scale metabolic networks of the individual species. Download FIG S3, PDF file, 0.05 MB. Copyright © 2019 Nielsen et al. 2019 Nielsen et al. This content is distributed under the terms of the Creative Commons Attribution 4.0 International license.

To further investigate which functions were affected across species, a gene set analysis was conducted, and significantly enriched gene sets were defined as either up- or downregulated. This allowed us to identify processes affected across the species (Fig. 2B; Table S1).

10.1128/mSystems.00012-19.9 TABLE S1 Gene set analysis results. Enrichment of GO terms and MetaCyc pathways for all six *Penicillium* species in complex medium relative to defined medium. Download Table S1, XLSX file, 0.3 MB. Copyright © 2019 Nielsen et al. 2019 Nielsen et al. This content is distributed under the terms of the Creative Commons Attribution 4.0 International license.

Only one process was conserved among the six species, namely, nitrate assimilation in the GO annotation, which was upregulated in CM, in agreement with the difference in the nitrogen source between the two media. Ammonium transport was upregulated in five species, based on GO terms, and similarly, ammonia assimilation and nitrate reduction were upregulated in four species, based on MetaCyc pathways. Additional upregulated biological processes included the siroheme and porphyrin biosynthesis GO terms in four species. No processes were significantly downregulated in all species, but in five of the species, the GO term copper ion transport and the MetaCyc degradation pathways of the branched-chain amino acids (BCAAs) leucine, isoleucine, and valine were downregulated. In four species, the MetaCyc pathways thiamine and 2-methylcitrate cycle (2MCC) were downregulated as well.

Previous studies have shown that *Aspergillus* species respond to hypoxia by upregulating BCAA metabolism and mitochondrial processes. The latter is seen as an increase in the content of metals, such as iron and copper cofactors, that are used in heme and porphyrin (22). Since we observed similar upregulated pathways, hypoxia might be a common driver of some of the metabolic changes observed in the species. In contrast, nitrate and ammonium assimilation as well as copper transport may be unrelated to hypoxia and, rather, may be a specific response highlighting the difference in nutrient sources and availability between the two media.

In general, few processes were statistically significantly differentially regulated in the majority of the species, indicating that despite the species being within the same genus and, hence, phylogenetically related, many of the responses were species specific rather than general. As a consequence of this, one should be careful when extrapolating regulatory information between species in a diverse genus, such as *Penicillium*.

### Expression and clustering of secondary metabolism in *Penicillium*.

Genome mining of the six *Penicillium* genomes revealed a total of 311 encoded secondary metabolite biosynthetic gene clusters (BGCs), and these were grouped into 42 gene cluster families (GCFs), consisting of similar BGCs (Fig. S4). Among these GCFs, 32 contained PKS, NRPS, NRPS-like, or PKS-NRPS backbone genes. Our recent annotation of *Penicillium* BGCs was updated using the same approach described previously (7) and allowed us to link six of the GCFs to a metabolic product. Among the 32 GCFs, 17 were differentially expressed in at least one species (as determined based on the expression level of the backbone genes), and these were approximately equally distributed between up- and downregulation (Fig. 2C).

10.1128/mSystems.00012-19.4 FIG S4 Overview of identified BGCs in the six *Penicillium* species. All BGCs (nodes) were grouped into gene cluster families (connected by edges). Download FIG S4, PDF file, 0.02 MB. Copyright © 2019 Nielsen et al. 2019 Nielsen et al. This content is distributed under the terms of the Creative Commons Attribution 4.0 International license.

**FIG 3:**
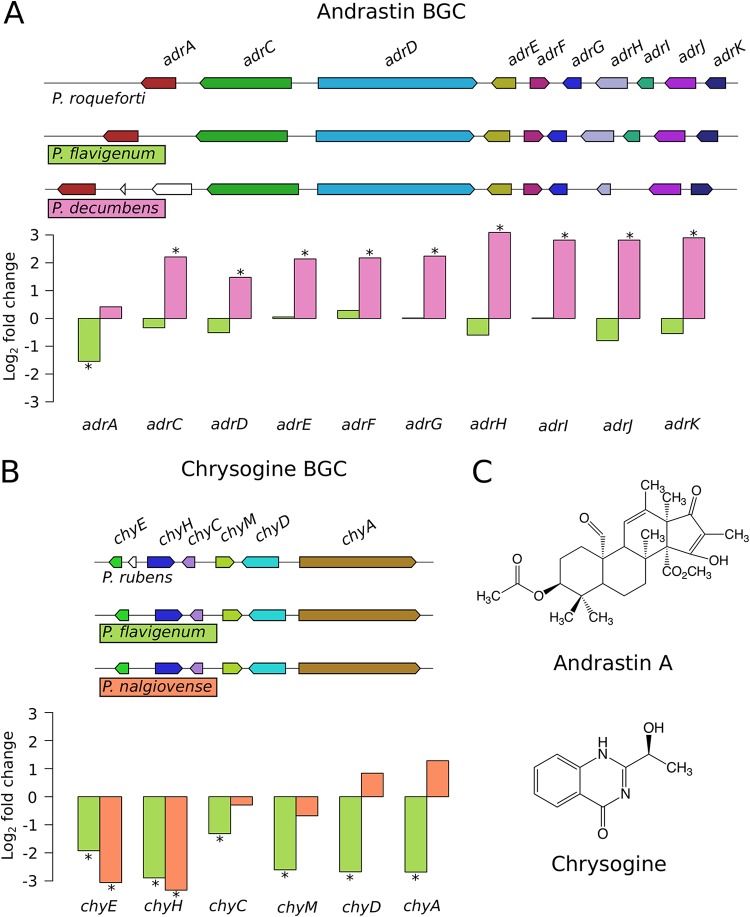
Expression of biosynthetic gene clusters (BGCs) in *Penicillium*. Andrastin BGC (A), chrysogine BGC (B), and chemical structures of the end products of the pathways (C) are shown. Bar plots represent the log_2_ fold change in expression levels in complex medium (CM) relative to defined medium (DM), and the colors of the bars correspond to the species. Asterisks denote genes that were significantly differentially expressed.

We correlated the expression levels of the annotated BGCs with the secondary metabolites detected in the fermentation media (Fig. S5). For four of the annotated BGCs, we detected the corresponding compound under at least one of the fermentation conditions. This included (i) andrastins for P. decumbens, (ii) chrysogines for P. flavigenum and P. nalgiovense, (iii) roquefortine/meleagrin intermediates for P. coprophilum and P. flavigenum, and (iv) fungisporin for P. coprophilum, P. nalgiovense, and P. flavigenum (14). We further looked into the expression of genes involved in previously characterized BGCs encoding andrastin biosynthesis in P. roqueforti (23) and chrysogine biosynthesis in P. rubens (24) (Fig. 3). All genes in the andrastin BGC in P. decumbens were upregulated, while chrysogine genes in P. flavigenum were downregulated. In the chrysogine BGC in P. nalgiovense, some genes were upregulated, while others were downregulated. Specifically, the genes *chyE* and *chyH* were significantly downregulated, while *chyA* and *chyD* were upregulated (Fig. 3). ChyA and ChyD catalyze the two first steps in the pathway, while ChyE and ChyH are thought to catalyze later steps (24). One explanation for observing these differences in expression could thus be a temporal transcriptional control based on when the individual enzymes are needed in the pathway cascade. It should be noted here that the RNA-seq data are indicative only for flux through the pathway and thus cannot be translated directly to increased production levels of the final compound. Quantitative metabolomics would be required to correlate gene expression with production levels and pathway fluxes.

10.1128/mSystems.00012-19.5 FIG S5 Secondary metabolite families detected in the bioreactor batch fermentations. The figure was generated based on previously published data (14). Download FIG S5, PDF file, 0.02 MB. Copyright © 2019 Nielsen et al. 2019 Nielsen et al. This content is distributed under the terms of the Creative Commons Attribution 4.0 International license.

### Coexpression modules in *Penicillium*.

In order to identify gene modules (groups of coexpressed genes) general to the *Penicillium* genus, the Pearson correlation coefficient (PCC) among all 3,782 single-copy core OGs as well as the 33 backbone genes identified in the 32 GCFs present in at least two species was computed. The correlations among these 3,815 OGs constituted a coexpression network from which nine coexpression subnetworks were extracted by removing connections between OGs based on absolute PCC cutoffs in the range of 0.1 to 0.9, which were denoted N1 to N9, respectively (Fig. S6). For each coexpression subnetwork, we identified highly connected gene modules using the ClusterONE algorithm (25), which allows for identification of overlapping modules, in agreement with the biological context where one gene product can have activities in multiple pathways. For larger networks, fewer but larger modules were found (Fig. S7), in agreement with previous observations (26), and a total of 59 significant modules were detected (*P < *0.1). To assess the overlap between these modules, the Jaccard index was computed based on module nodes (OGs), and this highlighted that many OGs were shared among multiple modules and, hence, represented different levels of resolution (Fig. S8). We removed highly redundant modules that shared the majority of nodes, and this reduced the total number of modules for the downstream analysis to 54.

10.1128/mSystems.00012-19.6 FIG S6 Overview of the pipeline for the coexpression analysis. Network N0 represents the global coexpression network, where each node represents a group of orthologous genes and edges represent Pearson’s correlation coefficient (PCC) weights. Nine subnetworks (N1 to N9) were generated by removing edges based on varying the PCC cutoffs. For each of these coexpression subnetworks, modules of highly coexpressed genes were detected using the ClusterONE algorithm (25). Download FIG S6, PDF file, 0.02 MB. Copyright © 2019 Nielsen et al. 2019 Nielsen et al. This content is distributed under the terms of the Creative Commons Attribution 4.0 International license.

10.1128/mSystems.00012-19.7 FIG S7 Overview of the number and size of the detected coexpression modules. (A) Number of modules in each of the subnetworks. Only significant modules were used for the further analysis. (B) Size of the significant modules from the different subnetworks. Download FIG S7, PDF file, 0.05 MB. Copyright © 2019 Nielsen et al. 2019 Nielsen et al. This content is distributed under the terms of the Creative Commons Attribution 4.0 International license.

10.1128/mSystems.00012-19.8 FIG S8 Similarity between detected coexpression modules. Similarity is calculated as the Jaccard index, based on the number of shared nodes between each module. Download FIG S8, PDF file, 0.04 MB. Copyright © 2019 Nielsen et al. 2019 Nielsen et al. This content is distributed under the terms of the Creative Commons Attribution 4.0 International license.

**FIG 4:**
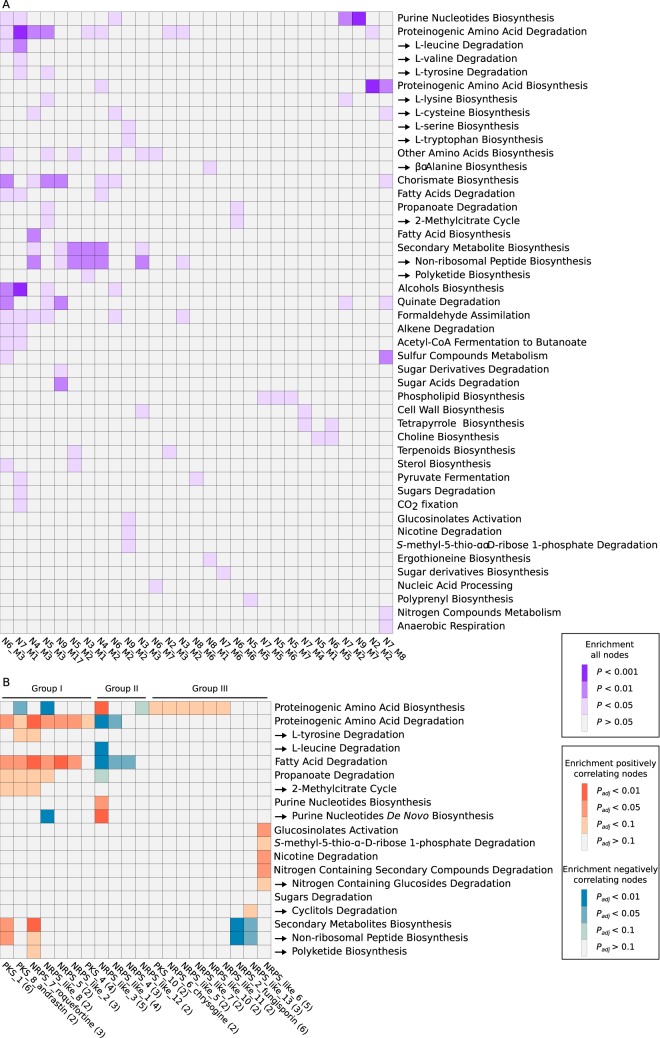
Enriched pathways in coexpression modules. (A) Pathway enrichment among orthologous groups in each coexpression module. Modules are denoted as N*X*_M*Y*, where *X* is the network number, and *Y* is the module number. (B) Pathway enrichment among orthologous groups connected to secondary metabolite backbone genes in the coexpression modules. Pathways were based on the third level of the MetaCyc annotation, while subpathways are indicated with arrows (the fourth level of the MetaCyc annotation). BGCs are denoted based on class, followed by an underscore and an identification number (e.g., PKS_1). Numbers in parentheses indicate how many of the six investigated species contain the BGC in their genomes. *P*_adj_, adjusted *P* value.

The 54 nonredundant coexpression modules were tested for enrichment of metabolic pathways using the MetaCyc annotation of metabolism, and a total of 29 modules were significantly enriched for at least one pathway (Fig. 4A). The most frequently occurring pathway was proteinogenic amino acid degradation, which was enriched in nine modules, and this included, in particular, degradation of leucine, valine, and tyrosine. Conversely, only three modules were enriched for amino acid biosynthesis genes, indicating that degradation processes were more predominant in the fermentations at the time of sampling. Biosynthesis of chorismate, a precursor of aromatic amino acids, was enriched in seven modules but did not overlap tryptophan biosynthesis, for which it is a precursor, thus indicating that chorismate might be used to a large extent for the synthesis of non-amino-acid metabolites, such as tetrahydrofolate, in fungi. There was no clear distinction between modules enriched for either biosynthetic or degradative pathways. For example, the network N7_M1 was enriched for both the degradation of amino acids and the biosynthesis of purine nucleotides (Fig. 4A).

Six modules were enriched for secondary metabolite biosynthesis. In three of these modules, amino acid degradation was also enriched, indicating a connection between the biosynthesis of secondary metabolites and the degradation of amino acids. In three of the modules, chorismate biosynthesis was enriched, which may be related to the biosynthesis of alkaloids derived from chorismate or to certain NRPs consisting of chorismate-derived amino acid building blocks.

### Expression of primary and secondary metabolism is tightly linked.

To further investigate interactions between primary and secondary metabolism, the connections of all secondary metabolite backbone genes were investigated in each of the 54 coexpression modules. The connections were divided into positive and negative correlations, and an enrichment analysis was conducted. All evaluated backbone genes were found in at least one of the coexpression modules, and among these, 20 were statistically enriched for MetaCyc pathways (Fig. 4B). Interestingly, the enriched pathways proved largely to revolve around the same pathways that were enriched in the coexpression modules as a whole (Fig. 4A) as well as some of the processes that were enriched across species in the gene set analysis (Fig. 2B). These pathways included degradation of amino acids, in particular, leucine and tyrosine; fatty acid degradation; and propanoate degradation via the 2-methylcitrate cycle (2MCC). Further, it was observed that, based on the correlated pathways, the backbone genes could be divided into three groups (Fig. 4B). Group I contained PKS and NRPS genes that were positively correlated with degradation processes, in particular, amino acid and fatty acid degradation, while they were negatively correlated with the biosynthesis of amino acids and purine nucleotides. Conversely, group II contained NRPS genes showing a reciprocal pattern of correlation, with a positive correlation to biosynthesis and a negative correlation to degradation pathways. Interestingly, the only biosynthesis process that was positively correlated to genes in group I was secondary metabolite biosynthesis. Group III contained PKS and NRPS genes that correlated with either high-level processes, such as amino acid biosynthesis, or specific pathways, such as cyclitol degradation. This group might represent secondary metabolite pathways that are activated only under very specific conditions.

**FIG 5:**
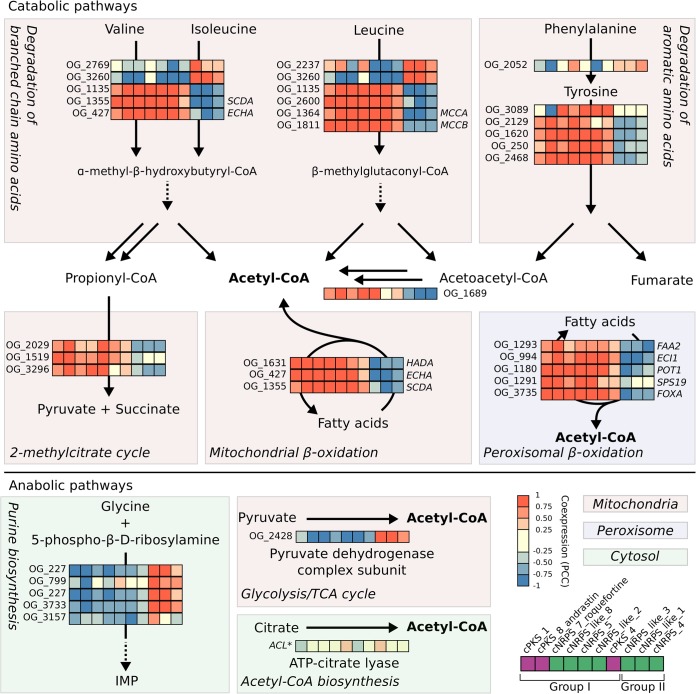
Main metabolic pathways correlating with secondary metabolite backbone genes. Heat maps represent backbone genes in the columns (as specified in the figure key) and orthologous groups (OG) responsible for the catalysis of the reactions in the pathways as rows. The figure key indicates PKSs in purple and NRPSs in green. Coexpression with genes marked with an asterisk was calculated for only five of the species. TCA, tricarboxylic acid.

We further investigated the pathways that were enriched in groups I and II to gain information about the specific correlations at the gene/reaction level (Fig. 5). This confirmed the sharp division between the backbone genes in group I and II as either positively or negatively correlated to degradation or biosynthesis. The products of the investigated degradation pathways proved largely to revolve around the mitochondrial acetyl-CoA pool, except for 2MCC, which is used for degradation of propionate, a toxic by-product of valine and isoleucine degradation. Purine biosynthesis was positively correlated with the three backbone genes of group II that were negatively correlated with degradation pathways, indicating that these backbone genes might be expressed under conditions where nucleotides are needed for growth. We further investigated other acetyl-CoA-generating reactions and observed that the genes in group II that were correlated with biosynthesis pathways were also correlated with one of the subunits in the pyruvate dehydrogenase complex. On the other hand, ATP-citrate lyase (ACL) did not show any clear correlation to any of the backbone genes. This suggests that pyruvate dehydrogenase activity, but not ACL activity, might contribute to the acetyl-CoA supply for secondary metabolism under growth conditions (Fig. 5).

## DISCUSSION

In this study, we have conducted a comparative transcriptome analysis of six *Penicillium* species in the stationary phase of bioreactor batch fermentations in a defined medium and a complex medium (14). The diversity among the species and the conditions under which they were cultivated were evident from the gene expression profiles, since a gene set analysis showed that only a few cellular processes were enriched across species. A coexpression network was constructed and allowed us to identify modules of genes that showed similarity in expression patterns. This enabled the identification of a metabolic signature which links central carbon metabolism to the production of secondary metabolites.

We found seven conserved secondary metabolite backbone genes (group I) with a strong correlation to the genes in mitochondrial and peroxisomal β-oxidation pathways. Previous studies have implied a fundamental role of both of these pathways in secondary metabolism. Disruption of the mitochondrial and peroxisomal β-oxidation pathways individually has been shown to reduce the production of the PK sterigmatocystin in A. nidulans (27). Further, mitochondrial β-oxidation is known to share an enoyl-CoA hydratase, *ECHA*, and an acyl-CoA dehydrogenase, *SCDA*, with the degradation pathway of the BCAAs isoleucine and valine (28, 29). Our data demonstrate a strong correlation between secondary metabolite backbone genes and genes encoding the degradation of all three BCAAs, including *ECHA* and *SCDA*, thus showing a close interplay between mitochondrial degradation of fatty acids and BCAAs. Both of these pathways yield acetyl-CoA, a precursor of PKs, as well as propionyl-CoA, and indicate that fatty acid and BCAA degradation might be the main sources of acetyl-CoA for PK biosynthesis under nutrient-limited conditions, such as in the stationary phase in DM, where the carbon source has been depleted. This is in agreement with previous metabolomics studies showing how disruption of the global transcriptional regulator of secondary metabolism, veA, leads to decreased PK biosynthesis as well as fatty acid and BCAA degradation in Aspergillus parasiticus (13). In addition to these pathways, we found that expression of genes involved in tyrosine degradation correlated with expression of secondary metabolite backbone genes, and this pathway has, to the best of our knowledge, not been linked to the precursor supply for secondary metabolism before. Intermediates from the degradation pathways of BCAAs could also possibly be utilized as building blocks for some PKs, as seen in bacteria (30, 31), as could propionyl-CoA (32). Propionyl-CoA is toxic and can be degraded via the 2MCC pathway, which was observed among the correlated pathways as well. Disruption of this pathway has been shown to have marked negative effects on the production of sterigmatocystin in A. nidulans, possibly because propionyl-CoA can block the active site of PKSs (33), thus highlighting the importance of detoxification of propionyl-CoA during PK production.

Among the seven backbone genes of group I that were seen to correlate with the above-mentioned acetyl-CoA-generating processes, only three were PKSs, while four were NRPSs (Fig. 5). The correlated pathways explain well how acyl-CoA units can be derived for PK biosynthesis during nutrient limitation but not why expression of NRPSs, which utilize amino acids as building blocks, was correlated as well. We did not observe any NRPS genes of group I to be correlated with amino acid biosynthetic pathways, so amino acids for NRPs are possibly derived from the breakdown of existing proteins instead of the *de novo* synthesis of amino acids. The directionality of this, i.e., if macromolecular breakdown leads to secondary metabolite formation or the other way around, cannot be determined from our data and would be interesting to investigate as a parameter for induction of secondary metabolite formation in an industrial setting. If, however, amino acids are synthesized *de novo*, the products from the degradation of BCAAs, i.e., acetyl-CoA, glutamate, and NADH, would be a favorable starting point for the synthesis of many amino acids. It could be speculated that upon induction of secondary metabolism, many PKs and NRPs are produced at the same time, and this could explain why we observed that NRPS genes also correlate with the degradation processes generating acetyl-CoA.

The three backbone genes of group II were anticorrelated with the acetyl-CoA-yielding degradation pathways and thus might encode secondary metabolites which are induced under nutrient excess. This is supported by the fact that they were correlated to nucleic acid and amino acid biosynthetic genes, which are active under growth conditions. During nutrient excess, pyruvate is produced through glycolysis and can then be converted into acetyl-CoA via the pyruvate decarboxylase complex. Our data suggest that the secondary metabolites that are produced during nutrient excess might use the acetyl-CoA generated via pyruvate decarboxylase activity as a building block (Fig. 5).

It is intriguing to speculate whether certain regulatory elements determine the observed grouping of backbone genes. We were not able to identify any conserved promoter motifs in the genes of BGCs defined in the same groups. This is likely due to the hierarchical structure of regulatory networks (34), resulting in BGC-specific transcription factor activation.

Taken together, our results suggest that the metabolism of filamentous fungi is tailored for the biosynthesis of secondary metabolites. During nutrient limitation, filamentous fungi direct metabolic flux through degradation pathways that generate the necessary precursor metabolites for the biosynthesis of secondary metabolites. We identified a metabolic signature that highlights the main pathways for precursor supply for secondary metabolite biosynthesis and found that expression of these pathways correlates with expression of certain PKS and NRPS genes. We further found that the precursor-generating degradation pathways were enriched in several coexpression modules, suggesting that major parts of metabolism are concerned with generating supplies for secondary metabolite biosynthesis. Even though many metabolic changes are also concerned with adapting the physiology to environmental changes, our data indicate that metabolism in filamentous fungi is tailored to meet the demands of secondary metabolite production.

As mentioned earlier in the Discussion, our data align well with observations on the metabolic regulation of secondary metabolism in *Aspergillus* species, which is why our findings likely can be extrapolated to other fungi as well. Recent findings for *Penicillium* (7, 16) and *Aspergillus* (35, 36) have shown a high genomic diversity and the adaptation of secondary metabolism to natural environments, but the supply of precursors for secondary metabolism might, on the contrary, be highly conserved. Our findings on the regulation of precursor-supplying pathways can thus aid in the design of metabolic engineering strategies to optimize the precursor supply for secondary metabolites in fungi, e.g., by overexpression of the precursor-generating pathways identified in this study. Although future experimental validation is necessary to fully map precursor-supplying pathways for secondary metabolites in fungi and to get a more detailed description of how metabolism is shaped toward secondary metabolite production, our findings provide a starting point in understanding how to manipulate metabolism for more efficient production of secondary metabolites.

## MATERIALS AND METHODS

### Cultivation conditions.

Samples for RNA-seq analysis were collected from cultivation experiments previously described (14). Briefly, six different *Penicillium* species (P. coprophilum [IBT 31321], P. nalgiovense [IBT 13039], P. polonicum [IBT 4502], P. decumbens [IBT11843], P. flavigenum [IBT 14082], and P. steckii [IBT 24891]) were cultivated in controlled bioreactors in a defined medium (DM) for *Penicillium* and in a complex medium (CM). Glucose was quantified by high-performance liquid chromatography, and CO_2_ concentrations were determined mass spectrometrically. Biomass samples for RNA-seq were withdrawn in the stationary phase, rinsed with ice-cold water through a Miracloth, and snap-frozen in liquid nitrogen until further analysis. Samples were collected from fermentations carried out in biological triplicate. All strains used in this study are available from the culture collection (IBT) at the Technical University of Denmark.

### Transcriptome analysis.

Cells from frozen biomass samples were disrupted using a TissueLyser LT disrupter (Qiagen), and total RNA was extracted using an RNeasy minikit (catalog no. 74104; Qiagen). DNA libraries for sequencing were prepared from the total RNA using Illumina’s TruSeq protocol and sequenced using an Illumina 2500 machine, yielding 99 nucleotide paired-end reads with an average insert size of 600 nucleotides. Raw RNA-seq reads were mapped to the individual *Penicillium* genomes using a documented work flow (37) based on the TopHat2 (version 2.0.9) (38) and HTSeq (39) programs. Both programs were run with default parameters. Gene-level statistics for CM versus DM were calculated using the DESeq2 program with default parameters, and differentially expressed genes were identified based on an adjusted *P* value cutoff of 0.05. For all downstream analyses, log-transformed expression levels were used.

### Determination of orthology and phylogeny.

The genome sequences of the six species were downloaded from NCBI, orthologous protein groups were identified using the orthoMCL algorithm (40) with default parameters, and these orthologous groups were used to define the core genome and the pangenome. Based on single-copy core genes, a concatenated maximum likelihood phylogenetic tree was reconstructed by using the RAxML program (41) and by applying a work flow previously described (7).

### Gene set enrichment analysis.

A gene set analysis was conducted based on a MetaCyc annotation (17) that was retrieved from previously published genome-scale metabolic models of the *Penicillium* species (16), and GO terms were annotated using the InterProScan (version 5.7-48) program (42). For the MetaCyc pathways, PKSs and NRPSs identified using the antiSMASH program (43) were added to the annotation of secondary metabolism. Gene set enrichment analysis of the DEGs was performed using the R package PIANO (44). Significant gene sets were identified based on a Benjamini-Hochberg-corrected *P* value cutoff of 0.05. Other enrichment analyses were conducted using the hypergeometric test implementation in R (phyper function).

### Identification and clustering of biosynthetic gene clusters.

BGCs were identified in the genomes using antiSMASH (version 4.0.0rc1 for fungi) (43), and the detected BGCs were clustered into gene cluster families (GCFs) using the BIG-SCAPE program (https://git.wageningenur.nl/medema-group/BiG-SCAPE). We tested various network clusterings in BIG-SCAPE, and finally, a cutoff of 0.6 for the overall score was selected on the basis of having similarity to previous clustering of *Penicillium* BGCs (7). Within GCFs, orthologous genes were identified using the MultiGeneBlast program (45), based on having 25% coverage and 30% identity.

### Coexpression network analysis.

Gene expression was correlated using the Pearson correlation coefficient (PCC). In addition, the single-copy orthologous genes were correlated with backbone genes (PKSs, NRPSs, NRPS-like, or PKS-NRPSs) present in at least two different species, as identified in the BGC clustering. The pairwise correlations of the expression of all 3,815 single-copy core genes constituted a weighted coexpression network with orthologous groups as nodes and PCC values as edges. This coexpression network was divided into nine subnetworks by applying different cutoffs for the PCC values (0.1 to 0.9), and the corresponding networks were denoted N1 to N9, respectively. For each of these subnetworks, correlation coefficients were converted to absolute values and normalized to distribute between 0 and 1 (minimum/maximum normalization). Highly connected clusters of genes, referred to as modules, were detected in the subnetworks using the ClusterONE algorithm (25). A total of 56 significant modules were identified using a *P* value cutoff of 0.1 for a *t* test assessing the connectivity within a module versus outside a module.

### Data availability.

The data sets supporting the conclusions of this article are available in NCBI’s Gene Expression Omnibus (46) repository under accession number GSE106983.
