# Supplementary figures and images for: Comparative Transcriptome Analysis Shows Conserved Metabolic Regulation during Production of Secondary Metabolites in Filamentous Fungi
Source: mSystems. 2019 Apr 16;4(2):e00012-19. doi: 10.1128/mSystems.00012-19 (PMC6469955; doi:10.1128/mSystems.00012-19)

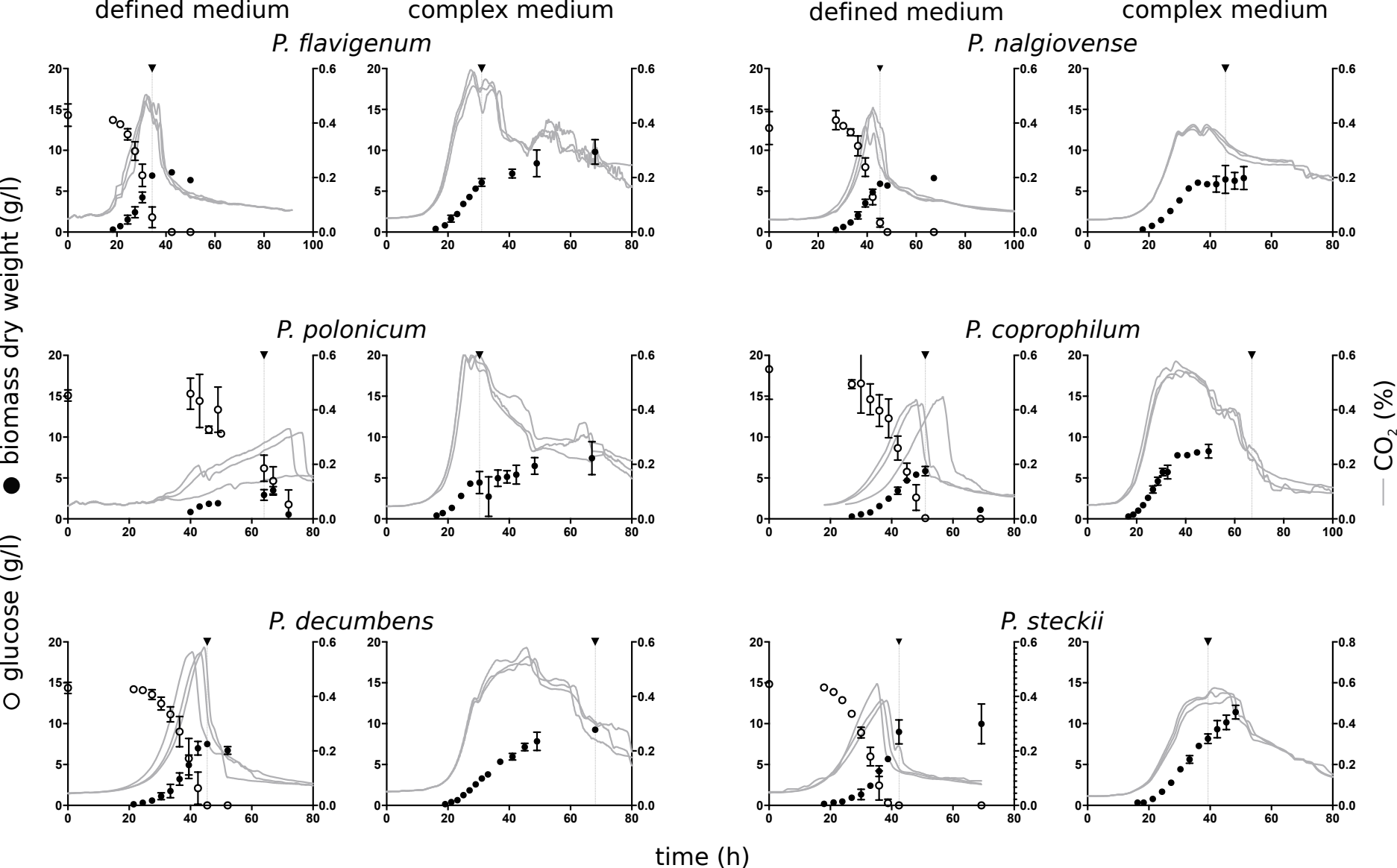

Supplement: FIG S1 [file mSystems.00012-19-sf001.pdf]

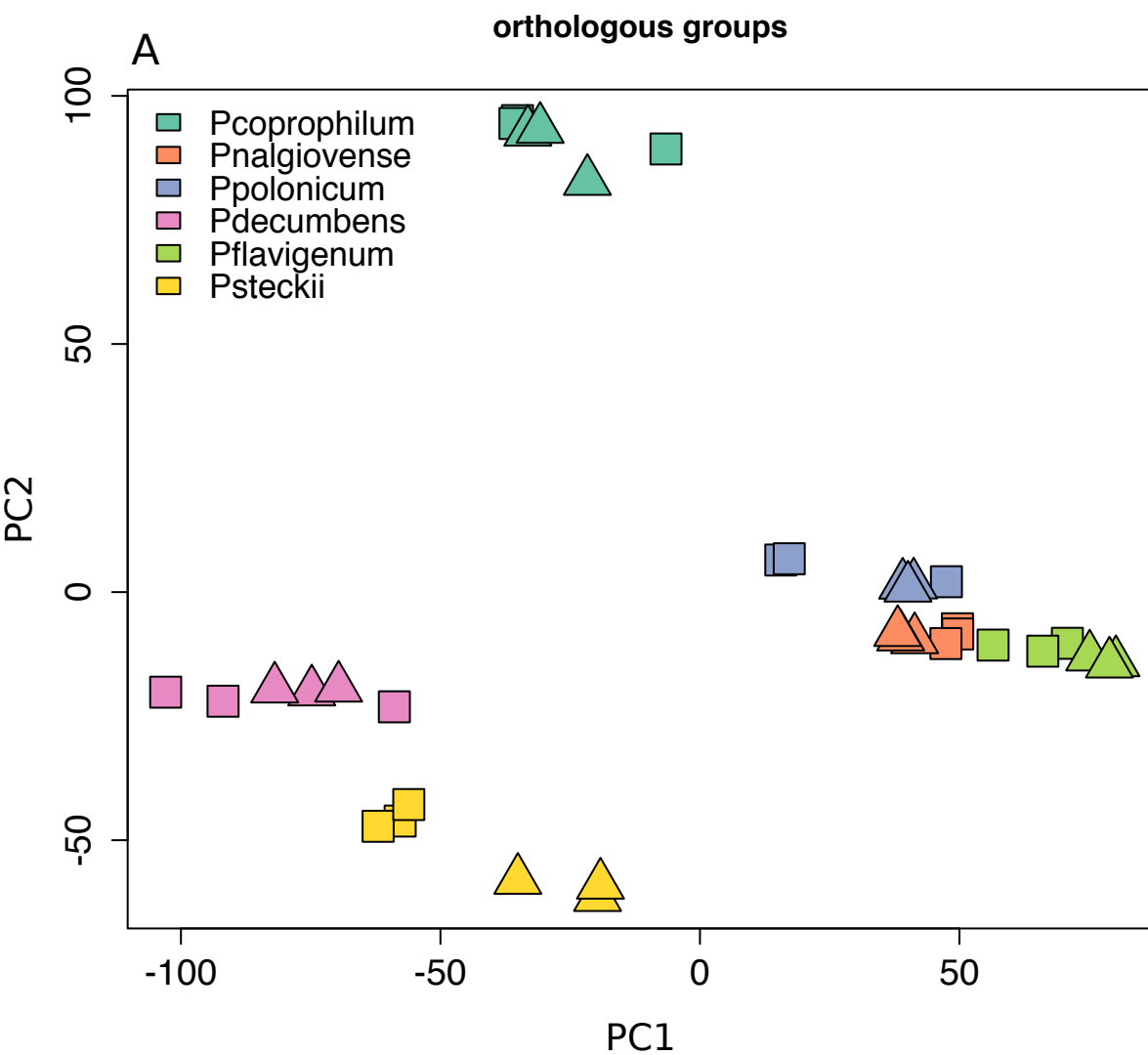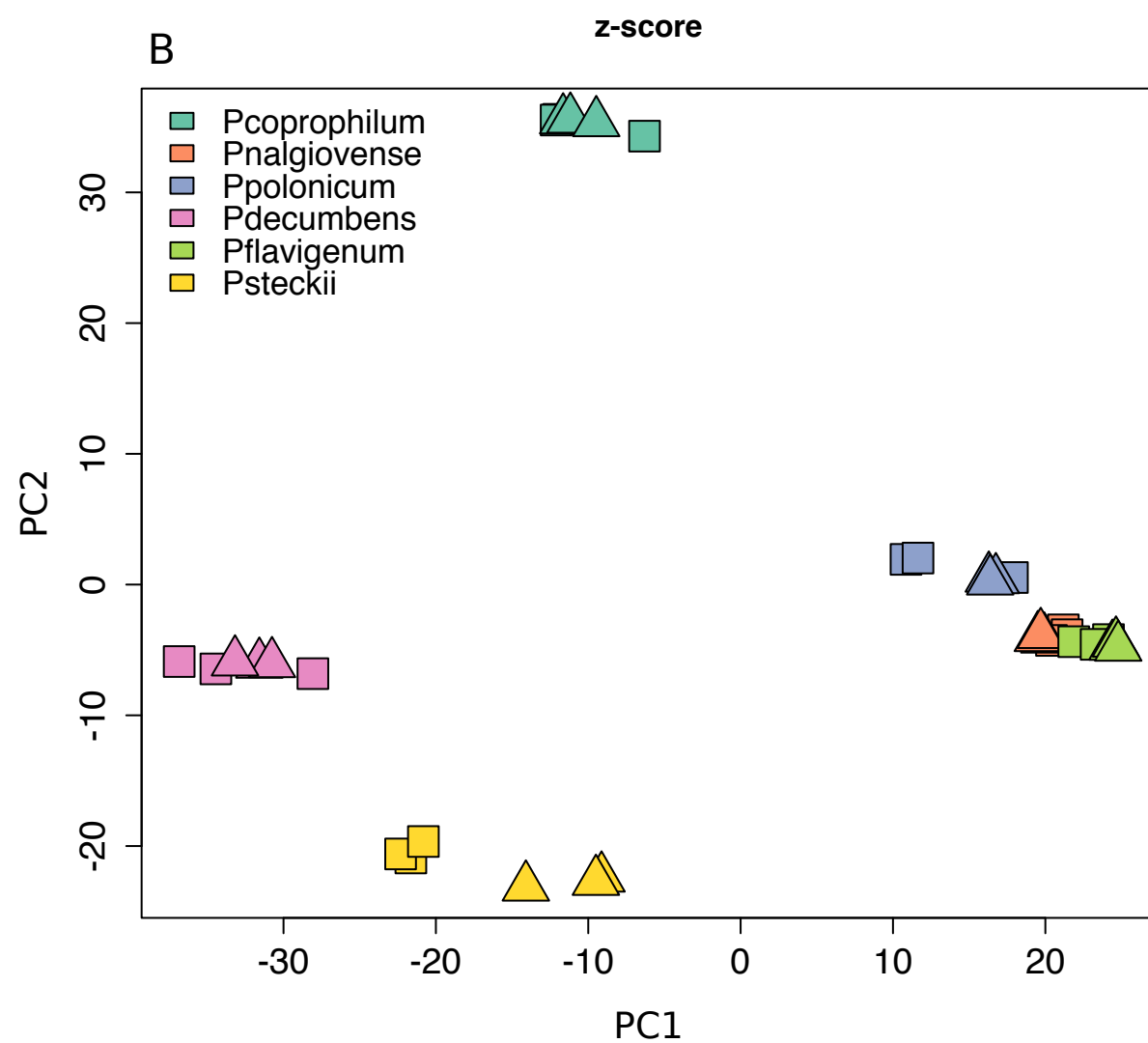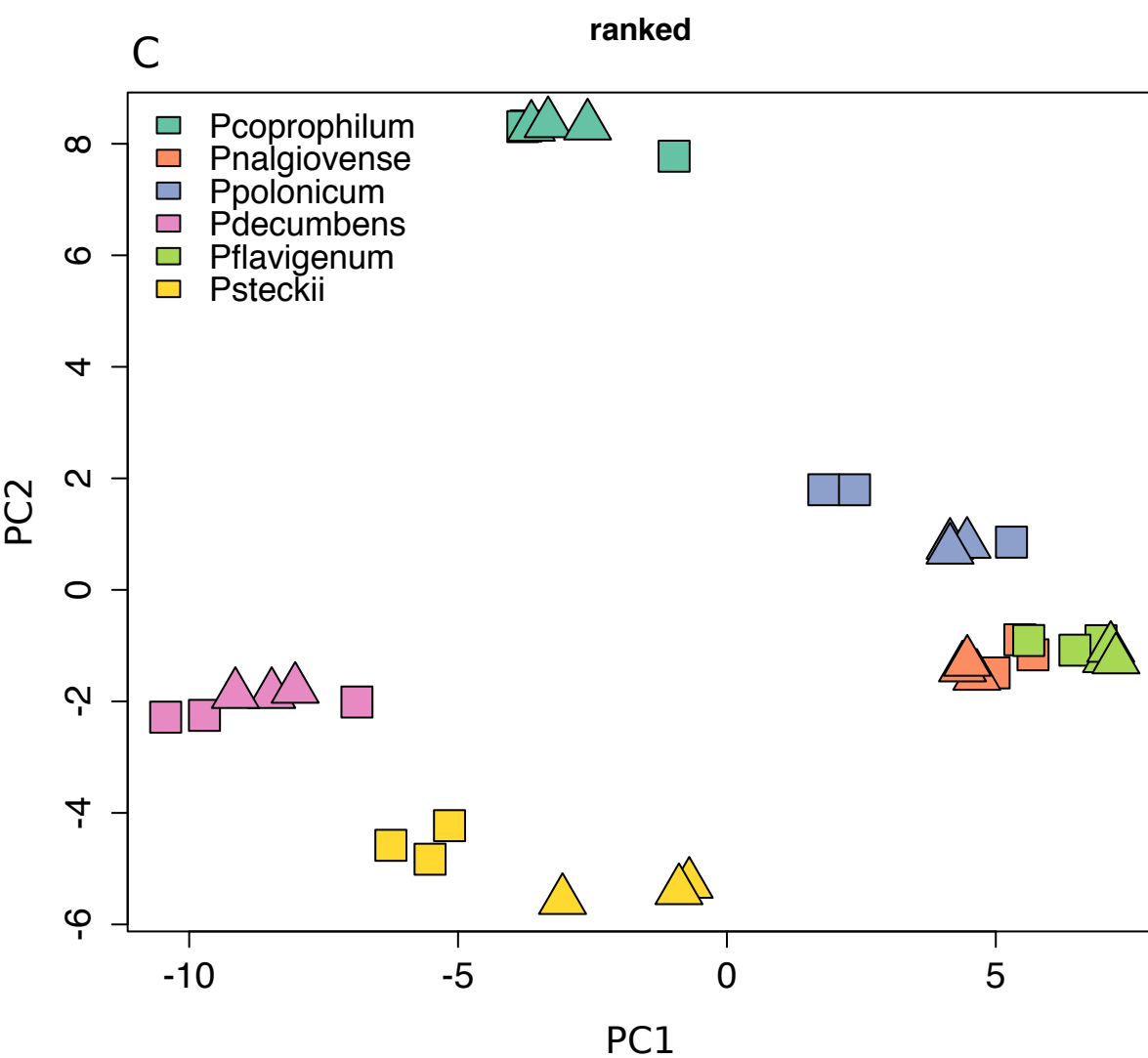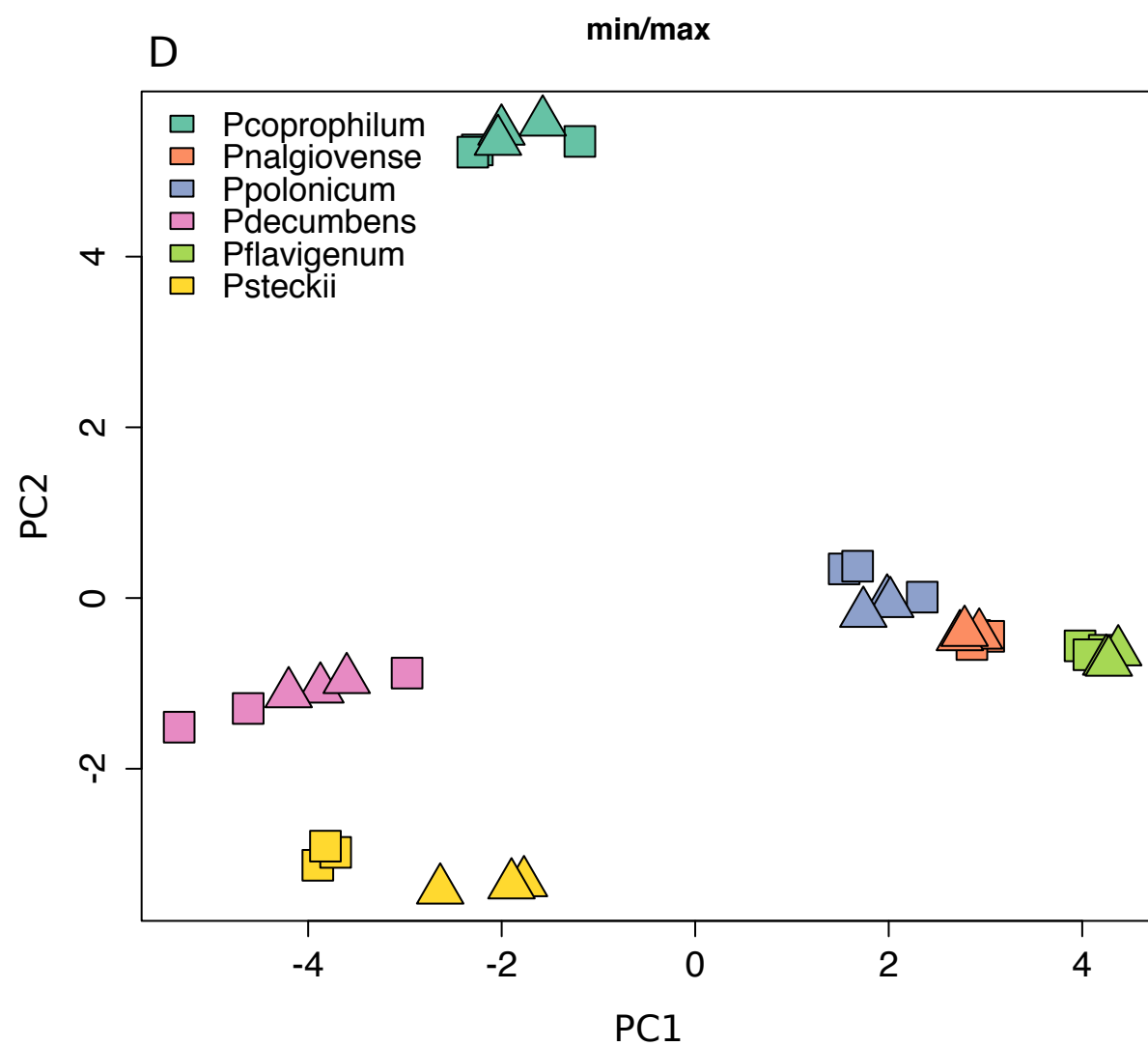

Supplement: FIG S2 [file mSystems.00012-19-sf002.pdf]

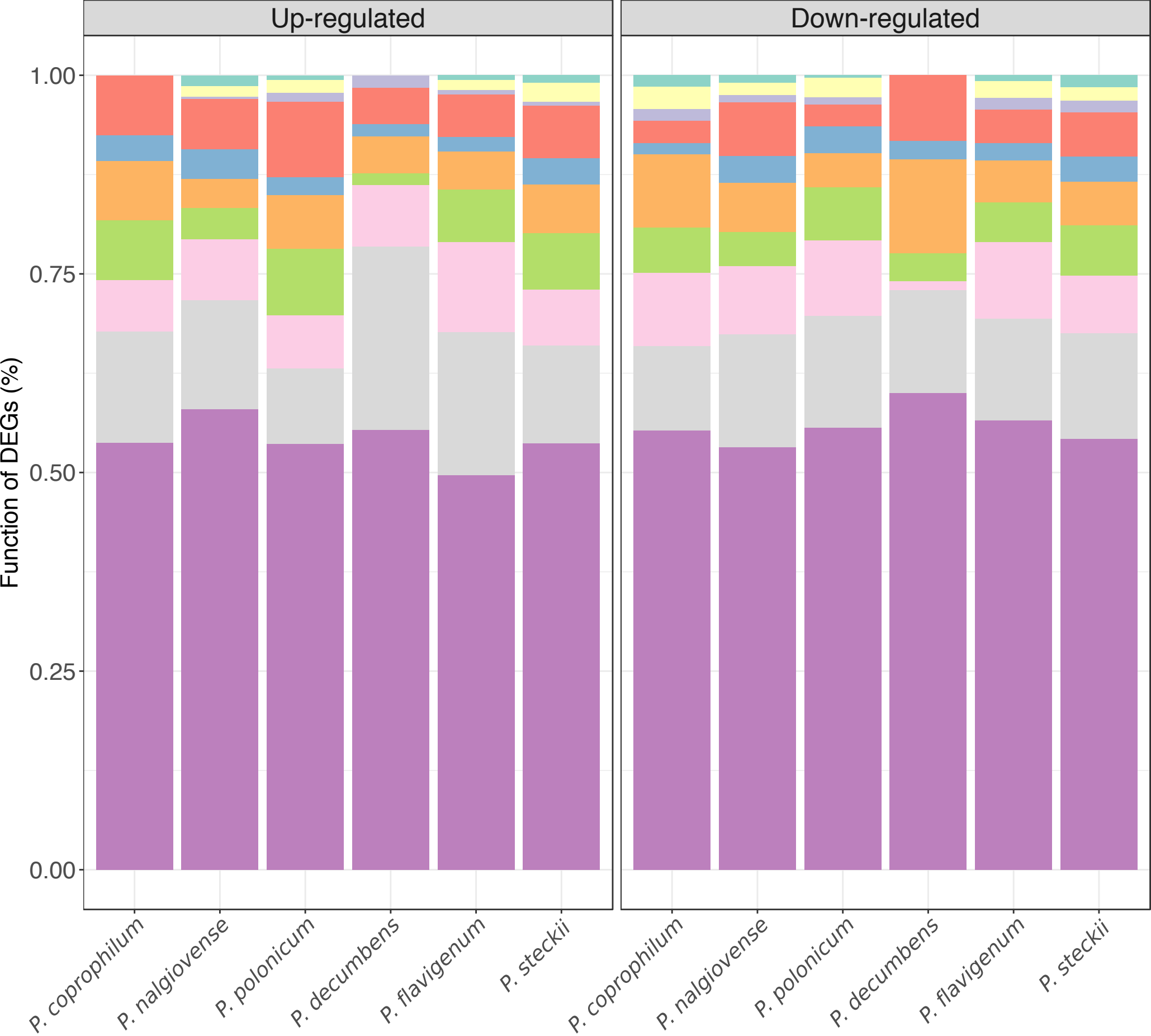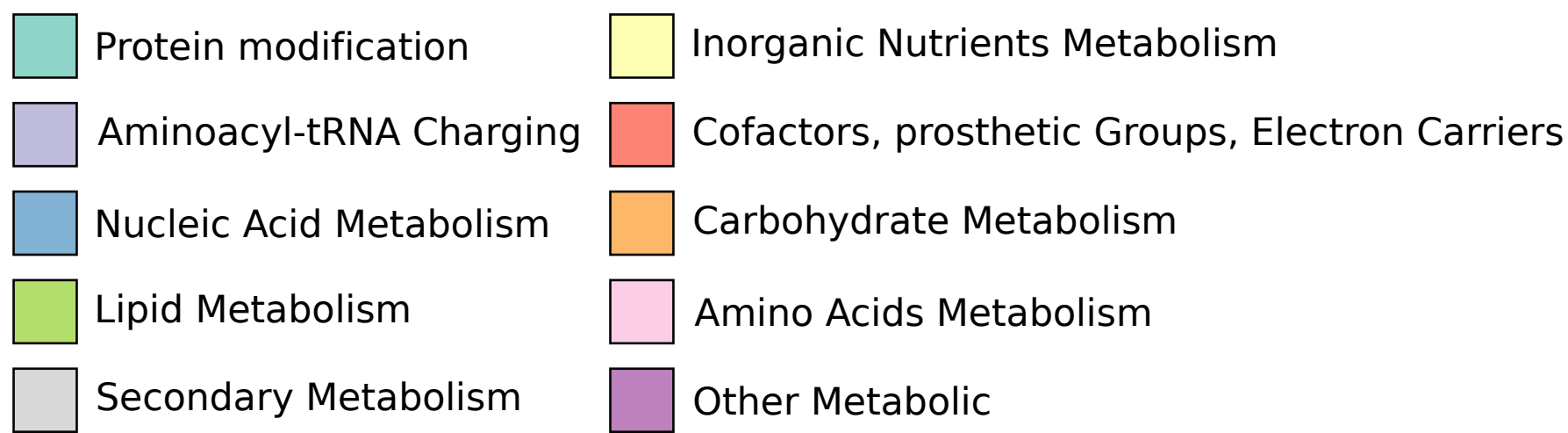

Supplement: FIG S3 [file mSystems.00012-19-sf003.pdf]

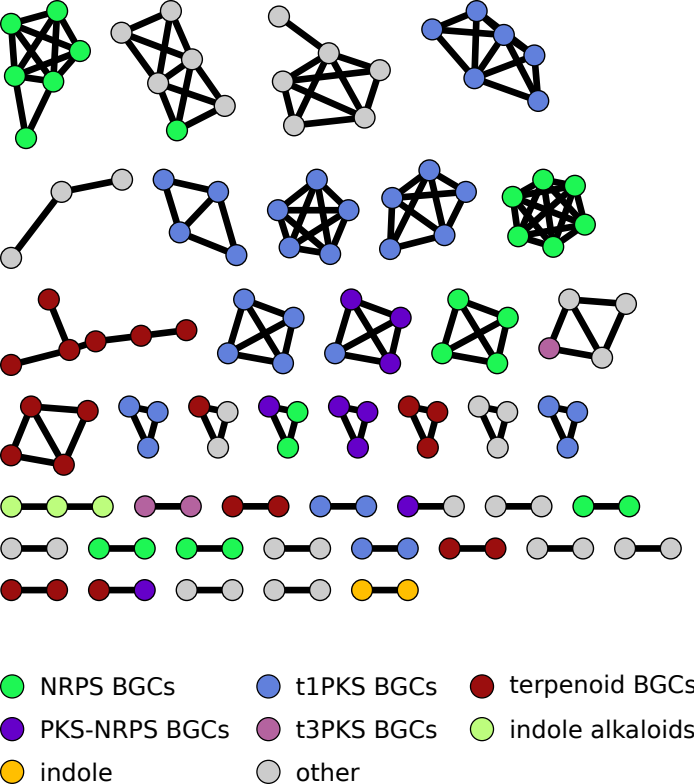

Supplement: FIG S4 [file mSystems.00012-19-sf004.pdf]

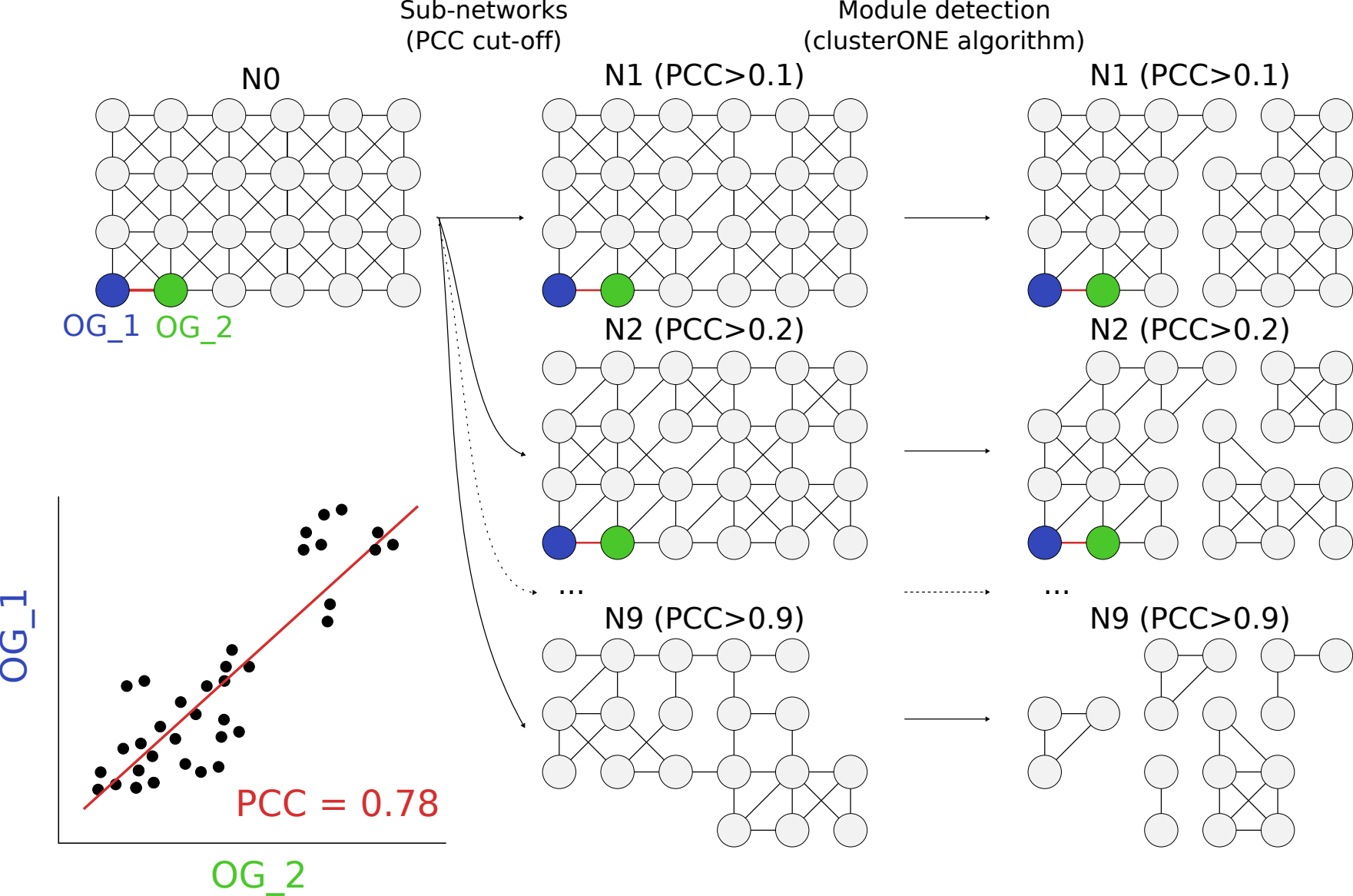

Supplement: FIG S6 [file mSystems.00012-19-sf006.pdf]

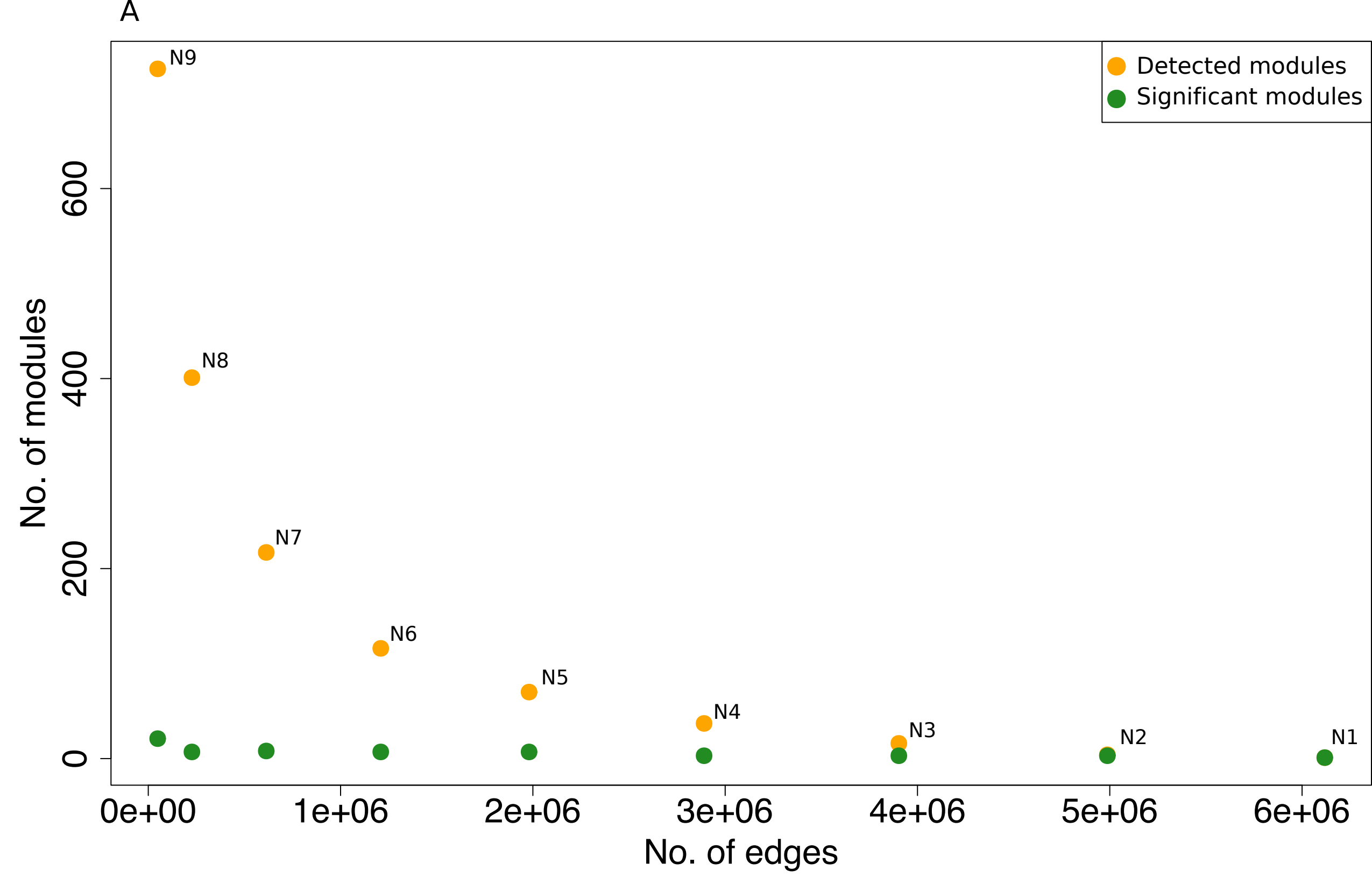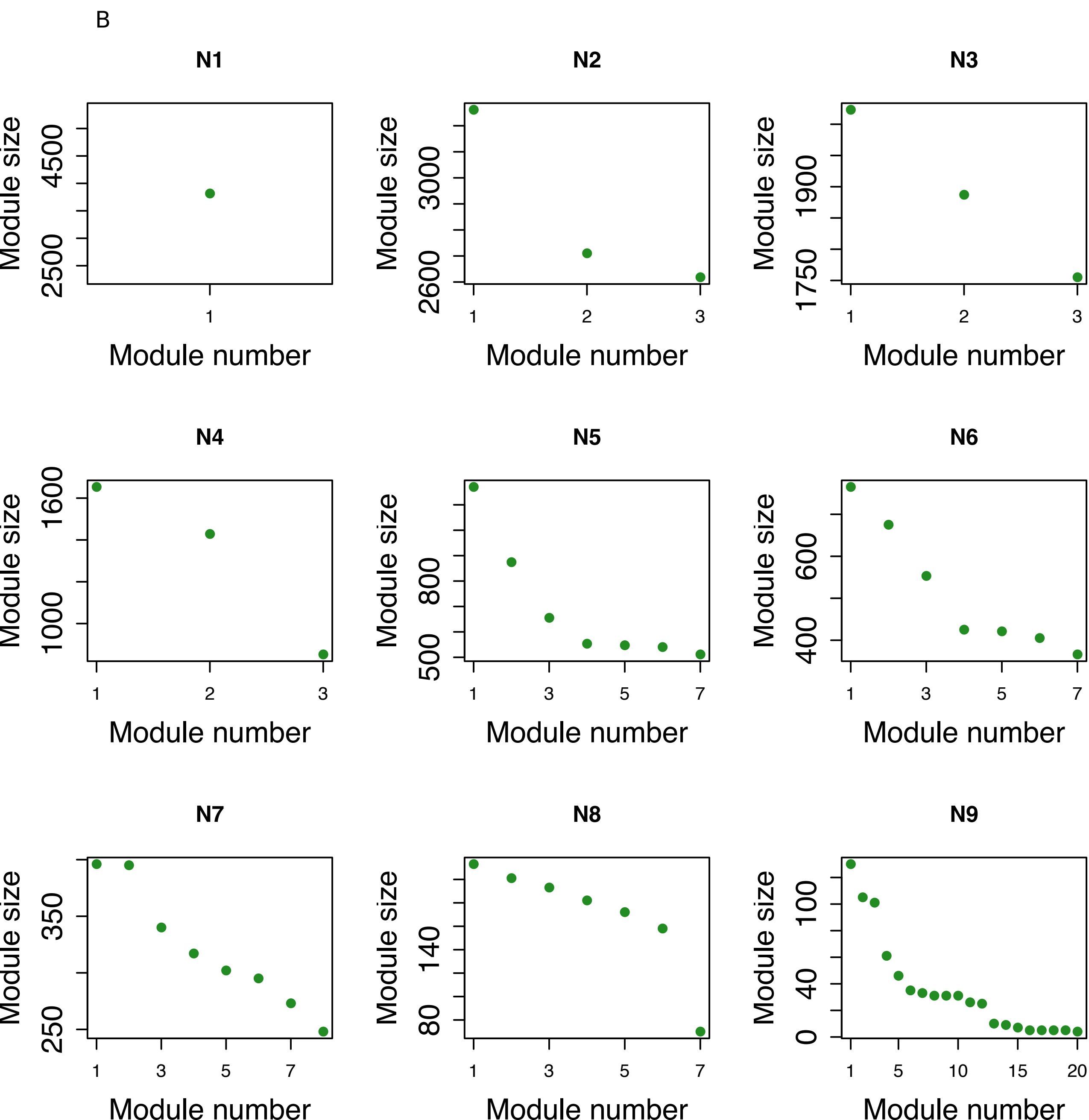

Supplement: FIG S7 [file mSystems.00012-19-sf007.pdf]

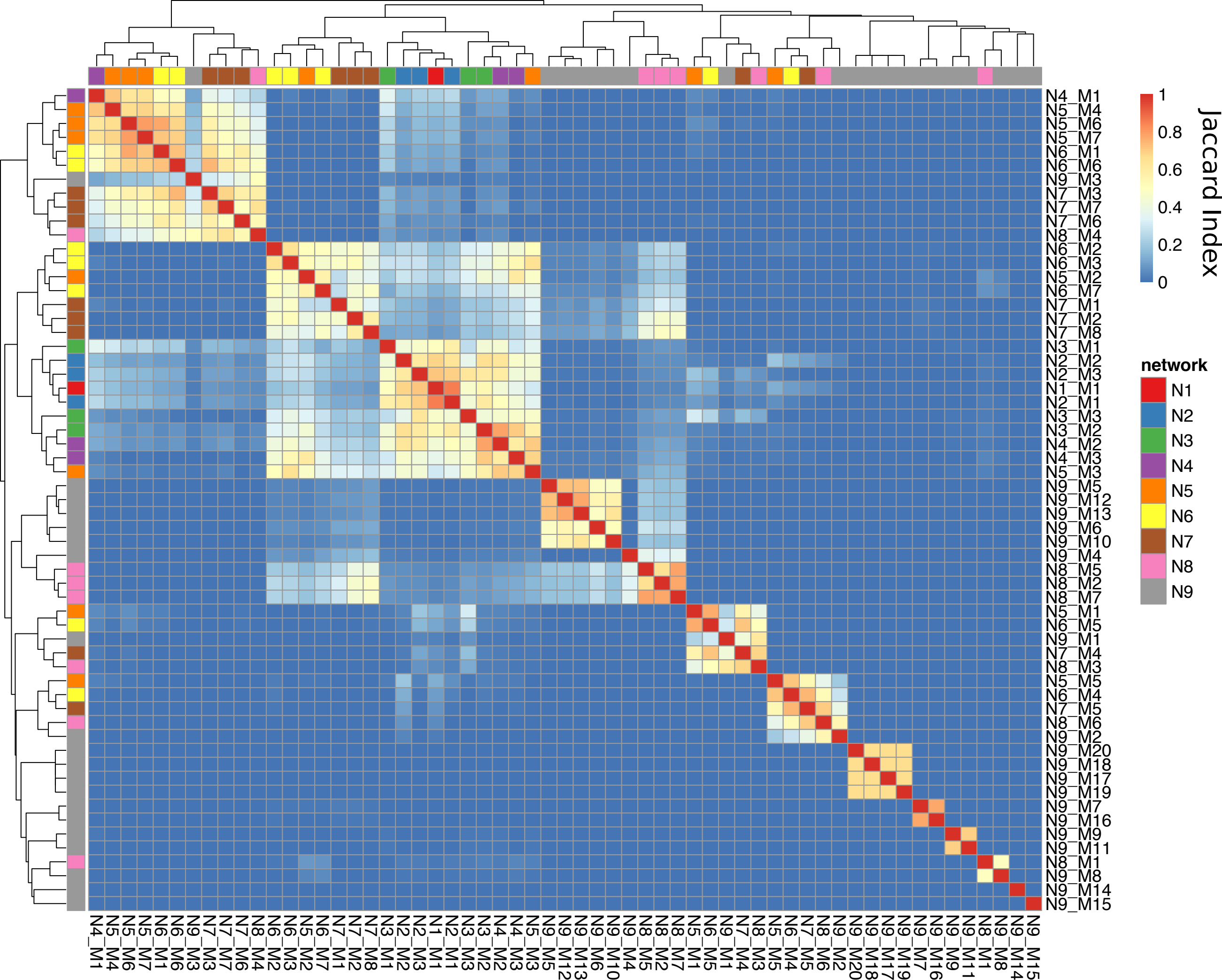

Supplement: FIG S8 [file mSystems.00012-19-sf008.pdf]
