# Supplementary material for: Comparative Transcriptome Analysis Shows Conserved Metabolic Regulation during Production of Secondary Metabolites in Filamentous Fungi
Source: mSystems. 2019 Apr 16;4(2):e00012-19. doi: 10.1128/mSystems.00012-19 (PMC6469955; doi:10.1128/mSystems.00012-19)
Supplement: FIG S5 [file mSystems.00012-19-sf005.pdf]

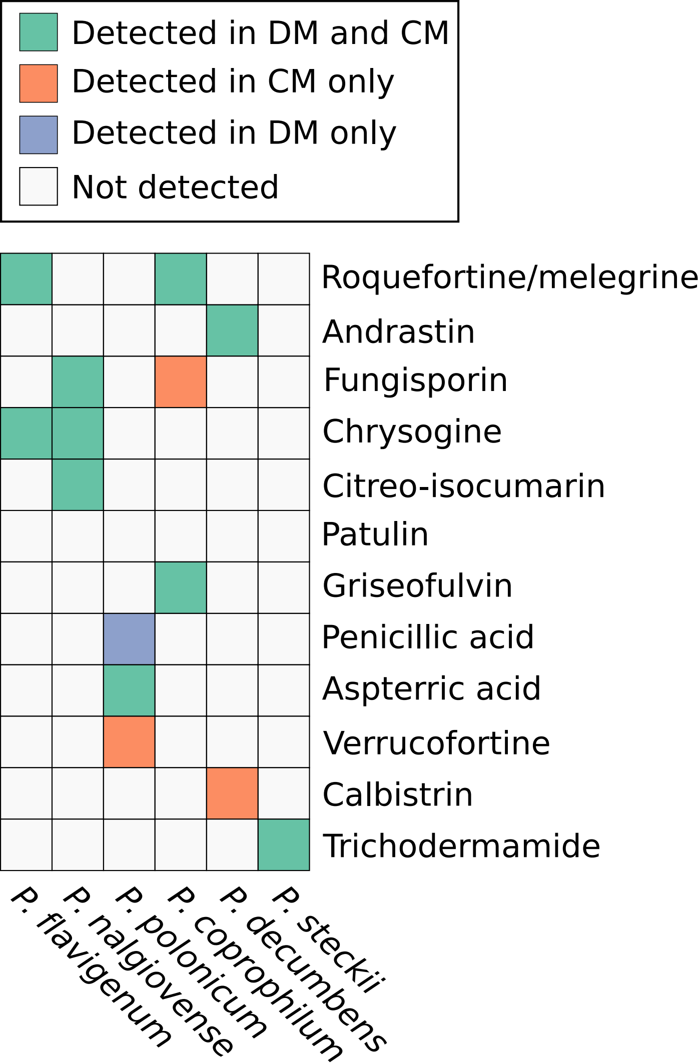
Detected in DM and CM
Detected in CM only
Detected in DM only
Not detected
Roquefortine/melegrine
Andrastin
Fungisporin
Chrysogine
Citreo-isocumarin
Patulin
Griseofulvin
Penicillic acid
Aspterric acid
Verrucofortine
Calbistrin
Trichodermamide
P. flavigenum
P. nalgiovense
P. polonicum
P. coprophilum
P. decumbens
P. steckii
